# Draft Genome Sequence of Strain R_RK_3, an Iron-Depositing Isolate of the Genus *Rhodomicrobium*, Isolated from a Dewatering Well of an Opencast Mine

**DOI:** 10.1128/genomeA.00864-17

**Published:** 2017-08-24

**Authors:** Burga Braun, Sven Künzel, Josephin Schröder, Ulrich Szewzyk

**Affiliations:** a Technische Universität Berlin, Chair of Environmental Microbiology, Berlin, Germany; b Max Planck Institute for Evolutionary Biology, Plön, Germany

## Abstract

*Rhodomicrobium* sp. strain R_RK_3 is an iron-depositing bacterium from which we report the draft genome. This strain was isolated from ochrous depositions of a mining well pump in Germany. The Illumina NextSeq technique was used to sequence the genome of the strain.

## GENOME ANNOUNCEMENT

A 16S rRNA gene sequence comparison of strain R_RK_3 revealed a 94% similarity to *Rhodomicrobium vannielii* ATCC 17100 (GenBank accession no. CP002292). The search was conducted using BLASTn (1) and EZBioCloud (2). However, determination of the most abundant taxon for the genome bin based on weighted scaffold length revealed 27.0% similarity to *Bradyrhizobium icense*.

The family *Hyphomicrobiaceae* contains the three phototrophic genera *Rhodoplanes* (3), *Blastochloris* (4), and *Rhodomicrobium* (5). Within the genus *Rhodomicrobium*, *R. vannielii* (5) and *R. udaipurense* (6) are currently the only recognized species. The draft genomes have been published for both of these species (7, 8). For *Rhodomicrobium vannielii*, the oxidation of ferrous iron has been described as a side reaction (9). The current work presents the third draft genome of a *Rhodomicrobioum* strain, isolated from a novel habitat, i.e., the well pump of a dewatering well that also showed iron-depositing activity.

Strain R_RK_3 was isolated from ochrous deposits from a dewatering well pump at the Hambach opencast mine, in the Rhenish lignite-mining area. Sequential dilutions of sampled ochrous material were spread on modified Leptothrix medium (10) (deionized water was replaced by sterile well water), and reddish-brown colonies were selected after incubation for 17 to 21 days at room temperature. The iron- and manganese-depositing activity of the isolate was verified by dissolving the colonies with oxalic acid (10%) as previously described by Schmidt et al. (11). Strain R_RK_3 is a small Gram-negative bacterium with a coccus shape of 2- to 4-µm length and a width of 1.0 µm.

Extraction of genomic DNA was done as previously described (12). The paired-end library was prepared by following the Illumina Nextera XT DNA library prep kit protocol. Genome sequencing was done on an Illumina NextSeq 500 sequencer using the NextSeq mid output kit v2 (300 cycles) by generating 74,681,576 raw reads. Demultiplexing was done with bcl2fastq v2.18.0.12, and quality filtering of raw reads was performed using Trimmomatic v0.36 (13), resulting in 48,202,264 filtered reads. Reads were then checked for ambiguous base calls and low complexity, employing the DUST algorithm (14), and filtered accordingly with an R script in Microsoft R Open v3.3.2 (15), followed by preassembly with SPAdes v3.10.0 (16) using default k-mer lengths up to 99 bp. Scaffolds ≥500 bp of this preassembly were subject to extension and second-round scaffolding with SSPACE standard v3.0 (17). Scaffolds ≥2,500 bp were assigned to genome bins by MetaBAT v0.32.4 (18), and functional annotation of draft genomes was performed with Prokka v1.12b (19). Tentative contig layout authentication (CLA) was conducted with Contig-Layout-Authenticator v1.0 (20) using the reference genome of *Rhodomicrobium vannielii* ATCC 17100.

The draft genome included 39 contigs with an *N*_50_ assembly quality of 226,044 and *L*_50_ of 10. The shortest scaffold was 2,822 bp, and the longest scaffold was 401,427 bp. The total size of the scaffolds was 5,786,497 bp with a G+C content of 65%. Annotation resulted in 39 contigs including 5,135 coding regions for 5,205 genes, 587 signal peptides, no clustered regularly interspaced short palindromic repeat (CRISPR) unit, 4 rRNAs (16S, 23S), 48 tRNAs, 1 transfer-messenger RNA (tmRNA), and 17 miscellaneous RNAs.

### Accession number(s).

This whole-genome shotgun project has been deposited at DDBJ/ENA/GenBank under the accession no. MWLK00000000. The version described in this paper is version MWLK01000000.
